# Tailoring the synergistic effect of integrated polypyrrole hydrogel on the adsorption activity of rice husk-based activated carbon (polypyrrole/activated carbon composite) for bisphenol-A and 4-chlorophenol: experimental and theoretical analysis

**DOI:** 10.3389/fbioe.2025.1556887

**Published:** 2025-03-21

**Authors:** Amna M. Farhan, Eman S. H. Khaled, Ahmed A. Abdel-Khalek, Ahmed M. El-Sherbeeny, Wail Al Zoubi, Mostafa R. Abukhadra

**Affiliations:** ^1^ Department of Chemistry, Faculty of Science, Beni-Suef University, Beni-Suef, Egypt; ^2^ Materials Technologies and their Applications Lab, Geology Department, Faculty of Science, Beni-Suef University, Beni-Suef, Egypt; ^3^ Industrial Engineering Department, College of Engineering, King Saud University, Riyadh, Saudi Arabia; ^**4**^ Materials Electrochemistry Laboratory, School of Materials Science and Engineering, Yeungnam University, Gyeongsan, Republic of Korea; ^5^ Applied Science Research Center, Applied Science Private University, Amman, Jordan

**Keywords:** activated rice husk, polypyrrole hydrogel, bisphenol, chlorophenol, adsorption, advanced isotherm

## Abstract

Rice husk-derived activated carbon was hybridized with polypyrrole hydrogel (Pyh), producing advanced nanocomposite (Pyh/AC). The composite was applied as an enhanced adsorbent for two forms of toxic phenolic compounds, particularly bisphenol-A (BSP-A) and 4-chlorophenol (4-CL). The adsorption studies were evaluated considering the synthetic effect of Pyh based on the criteria of statistical physics equilibrium modeling. The reported saturation adsorption capacities for BSP-A and 4-CL using Pyh/AC are 321.4 mg/g and 365.8 mg/g, respectively. These values are significantly higher than the estimated values for the hydrogel in separated form. The analysis of the steric properties validated the saturation of the composite with about 169.7 mg/g and 119.5 mg/g as active site density during the uptake of BSP-A and 4-CL, respectively. These values are higher than the estimated densities using Pyh (110.5 mg/g (BSP-A) and 99.3 mg/g (4-CL)), demonstrating the positive impact of the hybridization process in terms of surface area, porosity, and incorporated chemical functional groups. Furthermore, the capacity of each site on the structure of Pyh/AC to accommodate up to 3 molecules of BSP-A and 6 molecules of 4-CL displays the operation of multi-molecular mechanisms and the ordering of these adsorbed molecules vertically and in non-parallel forms. The adsorption energies, either based on classic (<21 kJ/mol) or advanced (<20 kJ/mol) isotherm studies, reflect the physisorption of the phenolic compounds on the surface of Pyh/AC. The composite also shows thermodynamically stable properties and the uptake reactions that occurred with exothermic, favorable, and spontaneous properties.

## 1 Introduction

Water quality and population security represent critical challenges in the modern era (Yang et al., 2022; Zourou et al., 2022). According to an urgent warning from the World Health Organization (WHO), by 2025, nearly half of the global population is projected to face water scarcity (Zourou et al., 2022; Arab et al., 2022; Mostafa et al., 2024). The rapid industrial growth observed over recent decades has exacerbated environmental issues, particularly water pollution, which poses significant risks to both human health and aquatic ecosystems (Ighnih et al., 2024; Kusuma et al., 2024). Among the various contaminants, endocrine-disrupting chemicals (EDCs) are exogenous molecules known to interfere with hormonal systems critical for development and reproduction in living organisms (Marlatt et al., 2021; Cambien et al., 2023). These chemicals are linked to numerous diseases and deformities. The European Union has identified over 900 compounds, including various phenolic species, as EDCs (Marlatt et al., 2021; Cambien et al., 2023; Czarny-Krzymińska et al., 2023).

Phenolic organic chemicals are widely recognized as highly hazardous water pollutants, primarily originating as byproducts from the production of insecticides, petroleum-derived products, and the timber industry (Gu et al., 2022). Among these, bisphenol A (BPA) [2,2-bis(4-hydroxyphenyl) propane] is a commonly synthesized phenolic compound classified as an endocrine-disrupting chemical (EDC) (Gu et al., 2022; Xing et al., 2022). BPA is extensively utilized in the manufacturing of various industrial products, including epoxy resins, fibers, electronics, healthcare materials, and leather tanning processes. Its presence as a micropollutant in aquatic environments has been linked to significant adverse health outcomes, such as cancer, congenital abnormalities, and hormonal imbalances, as well as increased risks of diabetes, cardiovascular diseases, and obesity (Gu et al., 2022; Tarafdar et al., 2022). Consequently, regulatory authorities recommend that BPA levels in human tissues remain below 50 μg/kg (Chen Z. H. et al., 2021), while water quality standards in countries like the United States and China mandate maximum BPA concentrations of 10 μg/L (Czarny-Krzymińska et al., 2023; Pruvost-Couvreur et al., 2021). Similarly, chlorophenols (CLs) are widely used in the production of chemical formulations, fungicides, and pesticides (Patel et al., 2022; Zarei, 2020). These compounds pose significant risks to marine ecosystems and human health even at low concentrations due to their environmental persistence, bio-accumulative nature, and pronounced biotoxicity (Dai et al., 2022; Taheri et al., 2023). Studies have shown that CLs exhibit mutagenic, carcinogenic, and genotoxic properties while resisting natural biodegradation, thereby contributing to long-term toxicity in the environment (Patel et al., 2022; Zarei, 2020; Hunge et al., 2022). Among these, 4-chlorophenol (4-CL) is extensively employed in the medicinal, petrochemical, pigment, and chemical industries (Lopes et al., 2024). The U.S. Environmental Protection Agency has classified 4-CL as one of the most hazardous environmental contaminants due to its toxicity, carcinogenic potential, and environmental stability (Taheri et al., 2023). As a result, the development of effective strategies and technologies for the removal of both BPA and 4-CL from contaminated environments is of critical importance.

The removal of 4-chlorophenol (4-CL) and bisphenol A (BPA) from the environment has been achieved through various methodologies, including photocatalytic degradation, biological treatments, adsorption techniques, and advanced oxidation processes (Caudillo-Flores et al., 2021; Shaban et al., 2023). Among these, adsorption using novel materials has been extensively studied and recognized as a cost-effective, efficient, reliable, user-friendly, and reusable method for the elimination of diverse water contaminants (Naat et al., 2021; Albukhari et al., 2021). The selection of an appropriate adsorbent depends on several critical factors, such as production costs, fabrication processes, precursor availability, adsorption efficiency, recyclability, adsorption kinetics, sustainability, selectivity, safety, and chemical reactivity (Fan et al., 2024; Abukhadra et al., 2020a). To address these requirements, researchers have conducted comprehensive evaluations to develop advanced adsorbents using readily available and economically viable raw materials, including resources derived from Earth’s natural reserves as well as agricultural and industrial waste products (Ifa et al., 2022; Sun et al., 2024). The utilization of well-established adsorbents sourced from such raw materials is strongly recommended due to their substantial environmental and economic advantages (Chen Y. et al., 2021).

Porous carbon is a versatile adsorbent material with extensive applications in the separation and purification of gas and liquid streams (Shaban et al., 2017a; Shen, 2022). Its structure consists of a microporous network, complemented by mesopores and macropores, which enhance the accessibility of adsorbate molecules to the carbon particles (Shaban et al., 2017a). The properties of porous carbon are determined by the type of precursor material used and the specific activation processes employed during its synthesis (Ma et al., 2023). Porous carbon can be produced from a wide variety of natural carbonaceous precursors, including coal, wood, corn, petroleum, peat, olive stones, almond shells, rice husks, and other biomass materials (Shen, 2022; Yan et al., 2023). Among these, rice husk is a significant agricultural byproduct generated during rice milling, accounting for approximately 20% of the total rice produced (Shaban et al., 2017a; AbuKhadra et al., 2020b). Composed of around 50% cellulose, 25%–30% lignin, and 15%–20% silica, rice husk is an abundant, underutilized material (AbuKhadra et al., 2020a). It is estimated that approximately 100 million tons of rice husk are produced annually in developing countries as a byproduct of rice production (Shaban et al., 2017a; Nzereogu et al., 2023). Rice husk exhibits several desirable properties, such as chemical stability, high mechanical strength, water insolubility, and widespread availability at minimal or no cost. These characteristics make rice husk an excellent raw material for the production of porous carbon, offering both environmental and economic benefits (Shaban et al., 2017a; Srivastava et al., 2023).

Previous studies have demonstrated significant improvements in the properties of carbon-based materials through chemical modification and surface treatment techniques (Abukhadra et al., 2022; Simate et al., 2016; Shaban et al., 2017b; Adel Sayed et al., 2023). These processes typically involve the activation of functional groups, the introduction of reactive chemical groups (particularly oxygen-rich groups), and the enhancement of surface area (Jawad et al., 2018; Jawad et al., 2020). One effective chemical activation method involves the oxidation of carbon materials, such as coal, using commonly employed acids. This technique has been shown to increase the electronegativity of the carbon surface and incorporate more efficient oxygenated functional groups into the material’s structure (Jawad et al., 2020; AlHammadi et al., 2022). Furthermore, the synthesis of polymer-based composites incorporating such chemically modified carbon materials remains underexplored, particularly for their application as adsorbents (Adel Sayed et al., 2023). It has been proposed that the integration of chemically activated carbon with polymeric materials could lead to advanced adsorbents with numerous active chemical groups functioning as adsorption sites.

Among the polymers investigated in recent studies, conductive polymers exhibit promising features, including high surface area, structural flexibility, remarkable stability, controlled pore size distribution, and the presence of various active groups, such as amino groups (Jawad et al., 2020; Ahmed et al., 2024a). Polypyrrole, a widely studied conductive polymer, belongs to the class of conjugated organic polymers (COPs) with a conjugated π-electron structure (Zhong et al., 2021). This polymer exhibits excellent adsorption capacity due to its high surface area and chemical reactivity, which arises from the abundance of nitrogen-containing functional groups. These groups display strong affinities for chemical ions through mechanisms such as hydrogen bonding and ion exchange (Zhong et al., 2021; Liu et al., 2020). Polypyrrole can be synthesized in various morphologies and forms, including polypyrrole hydrogel (Pyh), which features a three-dimensional (3D) interconnected structure. Polypyrrole hydrogel has garnered significant interest for various applications due to its unique properties, such as elasticity, mechanical durability, low density, exceptional swelling behavior, high redox activity, environmental safety, large surface area, tunable surface chemistry, and superior conductivity (Ahamad and Nasar, 2024; Yu et al., 2023; Wang et al., 2023). Additionally, its synthesis is cost-effective and straightforward, making it suitable for use as an adsorbent or as an active component in hybrid materials and composites. It is hypothesized that the development of a hybrid structure combining polypyrrole hydrogel and rice husk-based mesoporous graphitic carbon would result in an advanced, low-cost, multifunctional adsorbent with enhanced surface area, reactivity, and adsorption capacity for the removal of bisphenol A (BPA) and 4-chlorophenol (4-CL) from water.

The present study focused on the synthesis and characterization of a hybrid material composed of polypyrrole hydrogel and rice husk-derived mesoporous graphitic carbon (Pyh/AC) as a multifunctional adsorbent for the efficient removal of toxic BPA pollutants from water sources. Adsorption experiments were conducted synergistically, considering the behavior of the hydrogel (Pyh) as a separate phase. This included a comprehensive investigation of the variables influencing adsorption, as well as advanced modeling based on statistical physics theory. The study evaluated key parameters, such as the density of active sites, saturation uptake capacity, the number of adsorbed ions per site, thermodynamic functions, and energetic properties, providing a detailed understanding of the adsorption process.

## 2 Materials and methods

### 2.1 Materials

Rice husk (RH) was collected as agricultural waste from commercial rice mill in Sharqea Governorate, Egypt. NaOH (Sigma-Aldrich, 97% purity), nitric acid (Sigma-Aldrich, 69% purity), and phosphoric acid (Sigma-Aldrich, 85%) was applied during the activation steps and production of porous carbonaceous substrate. Pyrrole (98%, Sigma-Aldrich; Egypt), hydrochloric acid (≥37%, Sigma-Aldrich; Egypt), and potassium persulfate (99.99% Sigma-Aldrich) were employed in the preparation of polypyrrol hydrogel. Bisphenol-A (BPA> 99%; Sigma-Aldrich) and 4-chlorophenol (98%) were used as sources for the polluted solutions. These chemicals are all of analytical grade and are utilized without additional purification.

### 2.2 Synthesis of the polypyrrol hydrogel/activated carbon composite (Pyh/AC)

#### 2.2.1 Synthesis of activated carbon (AC)

**FIGURE 1:**
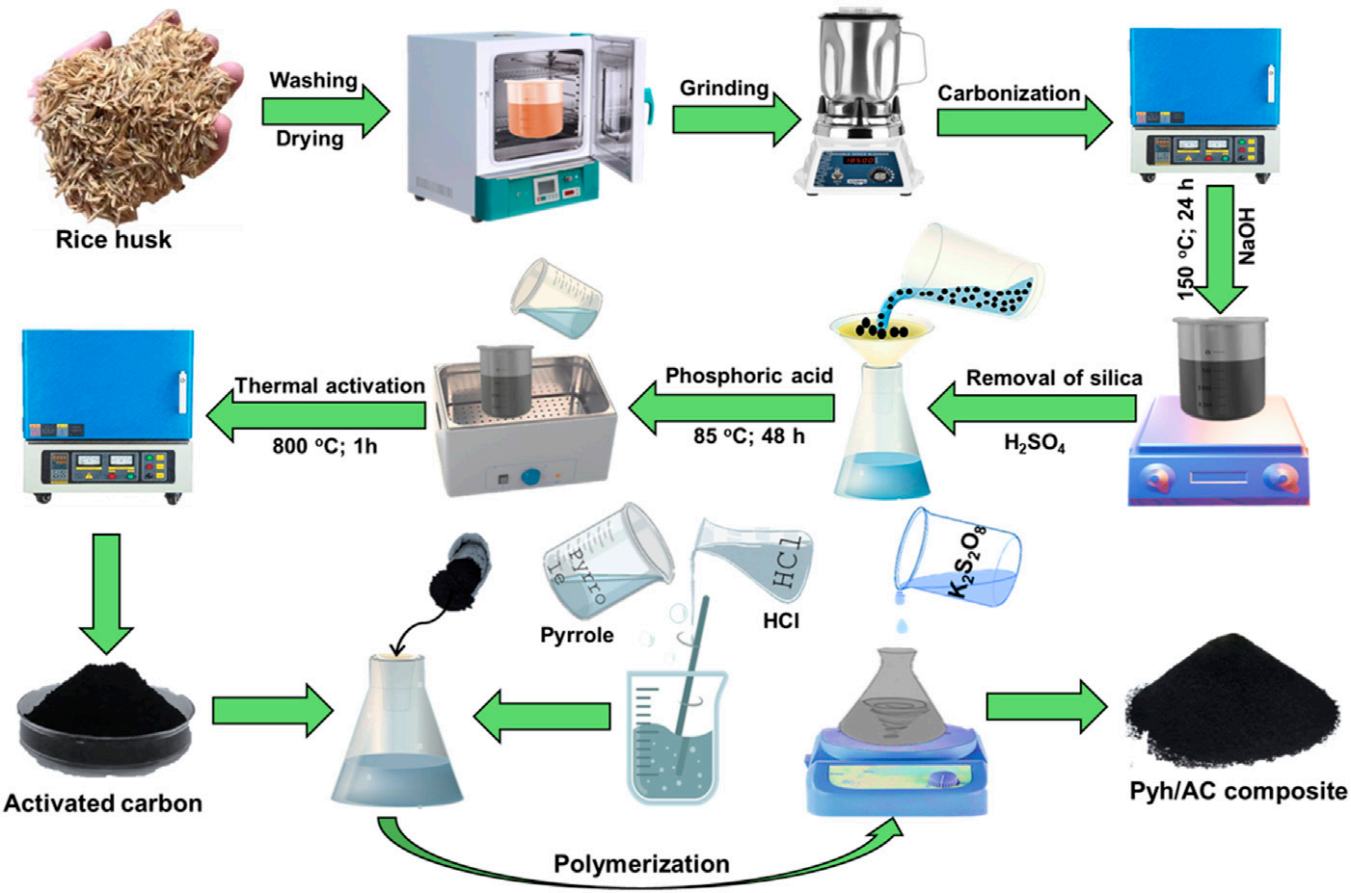
Schematic diagram for the synthesis of activated carbon and synthesis of Pyh/AC composite.

The starting rice husk was washed several runs using deionized water to remove any attached impurities before being subjected to drying step at 80°C for 24 h. The washed sample was then ground into micro-fractions of size 100–200 µm using traditional household blender. The ground RH (200 g) was then subjected to carbonization step for 3 h at 500°C using digital muffle furnace. The carbonized RH (CRH) was ground and pulverized inside NaOH solution (1 M; 300 mL) and homogenized for 24 h at 150°C. This step resulted in soluble sodium silicate solution containing suspension of silica free CRH which was obtained by filtration using Whatman filter paper. The obtained silica free CRH fractions (10 g) were homogenized within phosphoric acid (85% concentration) at adjusted ratio of 1 (CRH): 2 (phosphoric acid) and thermally treated with digital water bath for 28 h at 85°C. After that, the product was activated thermally at 800°C for 1 h. Finally, the synthetic CRH based activated carbon was cooled down washed, neutralized, and dried for additional 5 h at 80°C to be applied in the next synthesis steps (Figure 1).

#### 2.2.2 Synthesis of polypyrrol hydrogel/activated carbon composite (Pyh/AC)

The synthesis of the composite was performed by oxidation polymerization techniques. The pyrrole monomer (12.5 mL) was dissolved using HCl solution (0.66 mL) under stirring for 5 min. Then the AC fractions (10 g) were mixed with the pyrrole solution under continuous stirring for 30 min. After this step, 3.5 g of the oxidizing reagent (K_2_S_2_O_8_) was dissolved within 2.5 mL of distilled water and then added slowly as drops to the mixture under stirring for additional 30 min at the room conditions. Following the complete polymerization of the polypyrrol as hydrogel in composite with AC, the obtained gel was extracted and washed several runs using ethanol followed by distilled water. Finally, the composite was left for 24 h to be dried in the room temperature and labelled as Pyh/AC (Figure 1).

### 2.3 Characterization instruments

The crystallographic properties and mineral phases of the samples were analyzed using X-ray diffraction (XRD) patterns obtained with a PANalytical Empyrean X-ray diffractometer, operating within a 2θ range of 0°–70°. Fourier transform infrared spectroscopy (FTIR) was performed using a Shimadzu FTIR-8400S spectrometer, capable of detecting wavenumbers between 400 and 4,000 cm^−1^, while elemental composition and changes in functional chemical groups during the synthesis process were assessed through energy dispersive X-ray (EDX) analysis. The surface morphology of the fabricated materials was examined using a Gemini Zeiss Ultra 55 scanning electron microscope (SEM). To ensure enhanced conductivity during imaging, the samples were coated with a thin gold layer through sputter coating. Additionally, the porosity and specific surface area of Pyh/AC were evaluated using a Beckman Coulter SA3100 surface area analyzer. This evaluation involved degassing the samples to remove residual gases, followed by nitrogen adsorption-desorption isotherm analysis to determine surface area and pore characteristics.

### 2.4 Adsorption studies

The adsorption experiments for bisphenol-A (BPA) and 4-chlorophenol (4-CL) using polypyrrole hydrogel (Pyh) and the Pyh/AC composite were conducted as batch studies under varying experimental conditions. The investigated variables included pH (3–10), adsorption duration (30–1,440 min), initial phenol concentration (50–450 mg/L), and temperature (293–313 K). Additionally, three parameters were fixed across all experiments: solid dosage (0.2 g/L), solution volume (100 mL), and agitation speed. All experiments were performed in triplicate, and the average values were reported to ensure accuracy and reliability. At the end of each equilibration period, the solid adsorbents were separated from the treated solutions via filtration using Whatman filter paper. The residual concentrations of the phenolic compounds in the filtrates were analyzed using a high-performance liquid chromatography (HPLC) system (Merck/Hitachi) equipped with a Luna column (150 mm × 4.6 mm, Phenomenex, United States) featuring a pentafluorophenyl (PFP) stationary phase with a 5 µm particle size. The system included an L-7400 UV detector, L-7100 pump, Rheodyne 7725i injection valve, and a 20 µL sampling loop. The adsorption capacity (*Qe*) in mg/g was calculated using Equation 1:

$$Q_{e\;\left(\text{mg}/g\right)}=\frac{\left(C_{o}-C_{e}\right)V}{m}$$ (1)

where *C*_*o*_ and *C*_*e*_ represent the initial and equilibrium concentrations of phenolic compounds (mg/L), *V* is the treated solution volume (mL), and *m* is the adsorbent dosage (g). This calculation allowed for the quantitative assessment of adsorption performance under the studied conditions.

The adsorption kinetics and isotherm models for the uptake processes using Pyh and Pyh/AC were evaluated through non-linear fitting of the experimental data to the equations of the respective models (Supplementary Table S1). The performance of the classic models was assessed using the determination coefficient (*R* ^*2*^) (Equation 2) and Chi-squared (*χ* ^*2*^) (Equation 3), which were calculated to determine the accuracy of the fitting. Advanced isotherm modeling was further conducted based on the principles of statistical physics. This analysis involved non-linear fitting of the experimental data to the equations of the advanced models (Supplementary Table S1) and was evaluated using the correlation coefficient (*R* ^*2*^) and root mean square error (*RMSE*) (Equation 4). The analysis accounted for parameters such as *m′* (inserted data), *p* (experimental variables), *Qi*_*cal*_ (theoretical adsorption capacity), and *Qi*_*exp*_ (experimental adsorption capacity), providing a comprehensive understanding of the adsorption behavior and the predictive accuracy of the models.

$$R^{2}=1-\frac{\sum {\left(Q_{e,\exp}-Q_{e,cal}\right)}^{2}}{\sum {\left(Q_{e,\exp}-Q_{e,mean}\right)}^{2}}$$ (2)

$$\chi^{2}=\sum \frac{{\left(Q_{e,\exp}-Q_{e,cal}\right)}^{2}}{Q_{e,cal}}$$ (3)

$$RMSE=\sqrt{\frac{\sum_{i=1}^{m}{\left({Qi}_{cal}-{Qi}_{\exp}\right)}^{2}}{m^{\prime }-p}}$$ (4)

## 3 Results and discussion

### 3.1 Characterization

**FIGURE 2:**
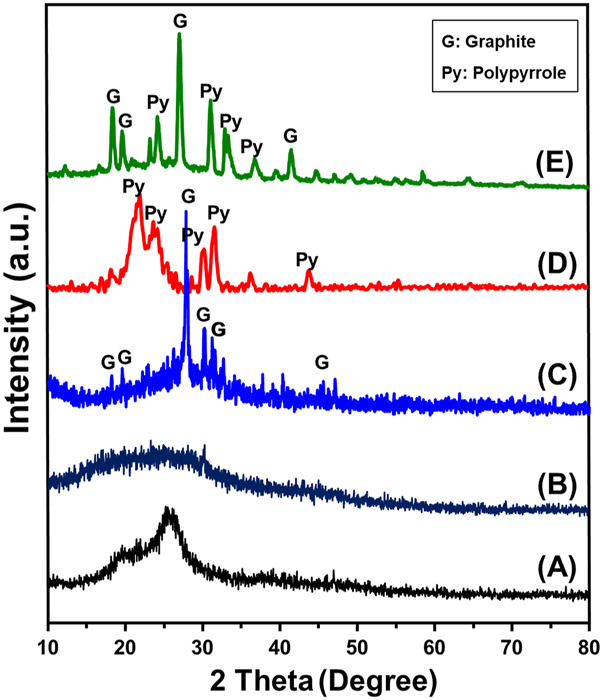
XRD patterns of rice husk **(A)**, carbonized rice husk (CRH) **(B)**, activated carbon (AC) **(C)**, polypyrrole hydrogel (Pyh) **(D)**, and synthesized Pyh/AC composite **(E)**.

The XRD patterns of the obtained composite as well as the integrated components during their different modification stages reflected the successful formation of graphitic carbon and integration of the Pyh (Figure 2). The included pattern of the raw RH reflects its amorphous properties with detectable broad peak around 2 Theta angle of 22^o^ which signifies the amorphous silica content (Figure 2A). After the alkaline treatment, the obtained pattern reflects the removal of the silica and the dominant broad peaks was observed around 2 Theta angle of 15^o^ demonstrating its amorphous carbon structure (Figure 2B). The pattern of the activated carbon reflected the conversion of the amorphous carbon precursors into crystalline graphite with significant peaks around 2Theta angles of 24.01^o^ and 26.58° with d-spacing 3.35Å and average crystallite size equal to 92 nm (JCPDS No. 25–0,284) (Figure 2C). The reported pattern of synthetic Pyh as separated phase confirms the successful polymerization of polypyrrol. This can be identified by the detected peaks around 21.8°, 23.9°, 30.4° and 31.5° (Rabia et al., 2023; Sharma et al., 2024) (Figure 2D). The recognized pattern of the final composite (Pyh/AC) confirms the successful integration between both the graphitic carbon and Pyy. The pattern shows complex peaks corresponding to the both component but with considerable deviation reflecting the chemical interaction and complexing between them (Figure 2E).

**FIGURE 3:**
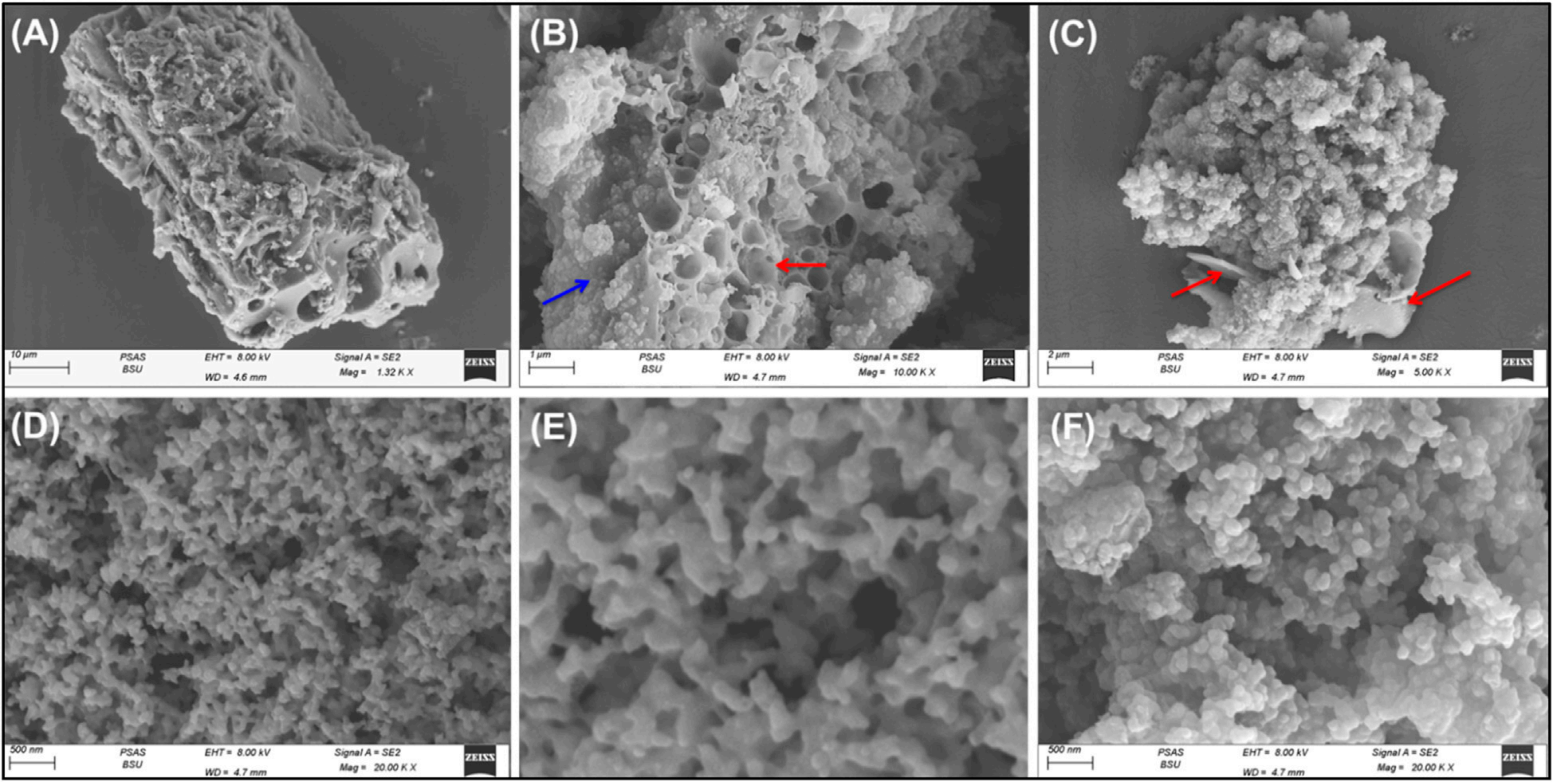
SEM images of activated carbon particles coated by Pyh **(A–C)** and focus image on the coated layer of Pyy hydrogel **(D–F)**.

The integration between the different components and the successful formation of the composite was also followed based on the SEM images (Figure 3). The activation step resulted in irregular particles of highly porous properties either connected pores or closed pores (Figures 3A, B). The Pyh appeared as coating layers on the surface of the activated carbon (Figures 3B, C). The high magnification images on the coated layers of Pyh reflected its formation as network structure with numerous intersected longitudinal grains forming highly porous matrix (Figures 3D, E). Other images declared the existence of Pyh particles as nanoaggregates of semi-spherical morphology (Figure 3F). These aggregates are interconnected with other producing interstitial nano-pores which induce the surface area and will be of significant impact on the adsorption properties of the composite.

**FIGURE 4:**
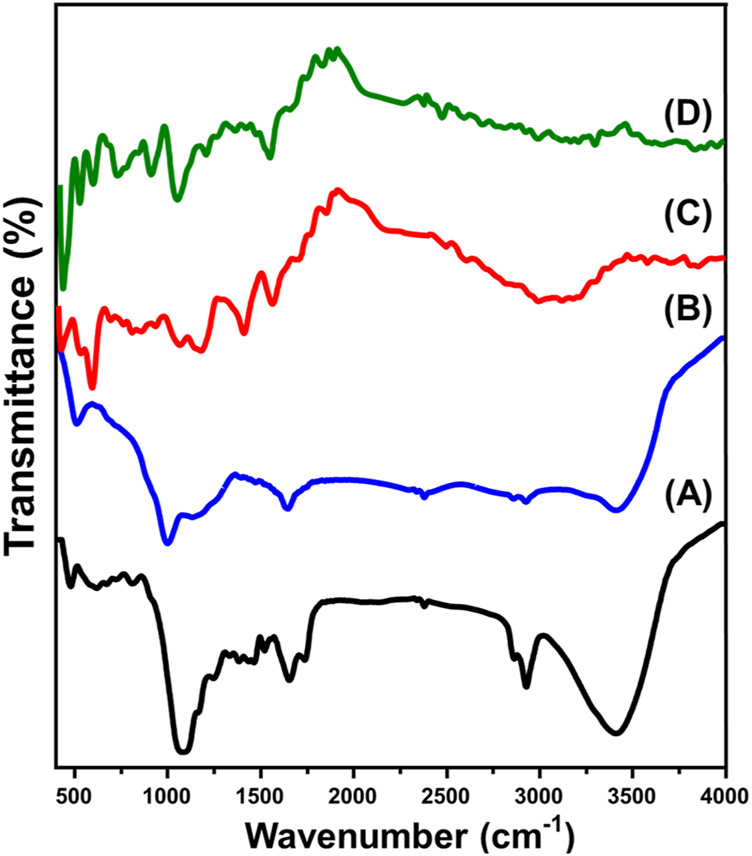
FT-IR spectra of rice husk **(A)**, activated carbon **(B)**, polypyrol hydrogel **(C)**, and Pyh/AC composite **(D)**.

The FT-IR spectra were used to follow the changes in the essential groups during the different modification and integration processes. The spectrum of the starting RH particulates declared the existence of its commonly reported chemical groups including O-H stretching (3,400 cm^−1^), aliphatic asymmetrical -CH_2_ (2,900 cm^−1^), aliphatic symmetrical -CH_2_ (2,800 cm^−1^), C=C or C=O bonding (1,640 cm^−1^), C–H bending (1,458 cm^-1^ and 1,376 cm^-1^), and SiO_2_ (bands from 570 cm^-1^ to 1,071 cm^−1^) (Figure 4) (AbuKhadra et al., 2020b; Zhang et al., 2023). After the activation process (CA), the recognized spectrum declared considerable reduction for several bands corresponding to the rice husk structure such as the marked bands around 1,458.2 cm^−1^ (C-H of methylene group) and 1,376.5 cm^−1^ (C-H in methyl group) alongside other bands around 1,071 cm^−1^, 800 cm^−1^, 611 cm^−1^, and 470 cm^−1^ which identify the existed silicon dioxide reflecting the complete removal of it after the alkaline modification process (Figure 4B) (Shaban et al., 2017a; Zawrah et al., 2023). The absorption bands observed in the range of 997–1,200 cm^-1^ in porous graphite can be attributed to the stretching vibrations of hydrogen-bonded P═O groups, O–C stretching in P–O–C (aromatic) linkages, and P═OOH groups (Figure 4B) (Shaban et al., 2017a). Additionally, the characteristic bands corresponding to nitrogen-containing functional groups in the synthetic activated carbon (AC) were identified at 1,548 cm^−1^, which were associated with C=N stretching and asymmetrical N=O stretching vibrations (Puziy et al., 2005).

The spectrum of the Pyh displays its functional chemical groups including amine group (N-H) (around 3,200 cm^−1^), stretching vibration of C-H (2,340 cm^−1^), C–N bending (1,680 cm^−1^), C=C stertching of pyrrole ring (1,551 cm^−1^), C=N bending (1,400 cm^−1^), stretching vibration of C-N+ (1,165 cm^−1^), plane vibration of C–H (1,060 cm^−1^), C=N^+^–C stretching (924 cm^−1^), out-of-plane ring deformation (661 cm^−1^), and C-H wagging inside the aromatic ring (794 cm^−1^) (Figure 4C) (Wu et al., 2021; Mahmood et al., 2024). Additionally, the dectablke bands within the frequency range from 700 cm^-1^ to 900 cm^-1^ signifies the C-H bonding in-plane and out-of-plane deformations (Sharma et al., 2024; Kanwal et al., 2023; Setshedi et al., 2024). Regarding the spcetrum of the composite, the recorded bands demonstarte the successful hybridization of AC particles with the Pyh (Figure 4D). This can be concluded based on the detection of complex bands correspondong to both AC and Pyh but at deviated positions reflceting the expected chemical interaction between them.

**FIGURE 5:**
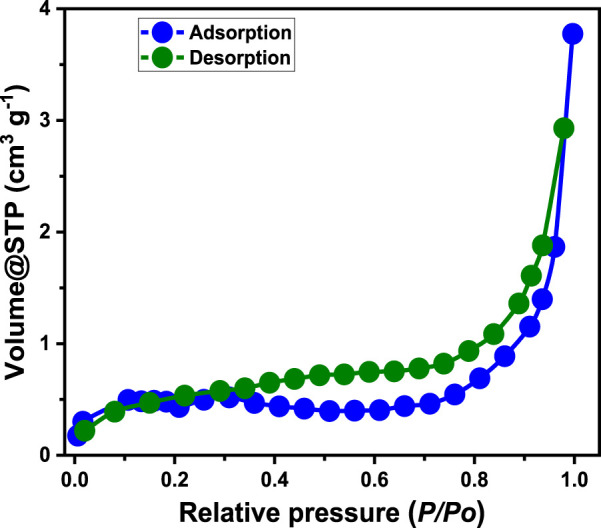
The nitrogen adsorption/desorption isotherm curve of Pyh/AC composite.

The textural characteristics of the synthesized Pyh/AC particles were analyzed using nitrogen adsorption/desorption isotherm curves (Figure 5). Based on the International Union of Pure and Applied Chemistry (IUPAC) classification, the observed curves correspond to type IV isotherms, with distinct hysteresis loops of type H3 (Xie et al., 2021). These features indicate the presence of mesopores (2–50 nm) within the Pyh/AC composite structure, which are attributed to non-rigid particle aggregation or slit-shaped pores (Xie et al., 2021). The Pyh/AC composite exhibits an average pore diameter of 9.5 nm and a specific surface area of 181.4 m^2^/g. These favorable textural properties highlight the material’s potential for effective use as an adsorbent for water contaminants or as a heterogeneous catalyst in chemical processes.

### 3.2 Adsorption results

#### 3.2.1 Effect of pH

**FIGURE 6:**
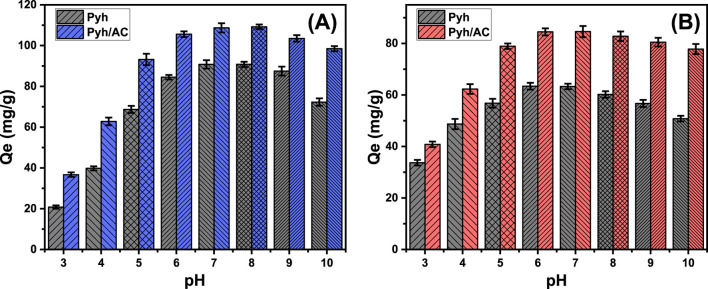
The influence of the solutions pH on the uptake of BSP-A **(A)** and 4-CL **(B)** by Pyh and Pyh/AC.

The pH of the aqueous solution plays a critical role in determining the surface charge distribution of the Pyh and Pyh/AC materials as well as the ionization behavior of water-soluble compounds (Jiang et al., 2020). The influence of pH on adsorption was evaluated across a range of pH values (3–10) under controlled conditions: contact time of 120 min, temperature of 293 K, solution volume of 100 mL, metal concentration of 100 mg/L, and adsorbent dosage of 0.2 g/L. A notable increase in the adsorption capacity of bisphenol (BSP-A) molecules was observed as the pH increased from 3 (Pyh: 20.8 mg/g; Pyh/AC: 36.7 mg/g) to 8 (Pyh: 90.6 mg/g; Pyh/AC: 109.2 mg/g) (Figure 6A). However, further increases in pH beyond 8 resulted in a significant decline in adsorption efficiency (Figure 6A). These results demonstrate that Pyh and Pyh/AC are effective adsorbents for BSP-A removal within the pH range of 6–9, which aligns with the industrial effluent standards recommended by the US EPA (Vivas and Cho, 2021). This pH-dependent adsorption behavior can be explained by the speciation of BSP-A and the surface charge characteristics of Pyh and Pyh/AC. BSP-A molecules exhibit two pKa values (9.6 and 10.2) (De Farias et al., 2022). In acidic to mildly basic conditions (pH 5–8), BSP-A primarily exists in a neutral form, facilitating its adsorption by electrostatic attraction onto Pyh and Pyh/AC surfaces. The highest adsorption efficiency occurs near the point of zero charge (pH_pzc_) of the materials (pH 7.6 for Pyh and pH 8.2 for Pyh/AC), where the surface charge is neutral, enhancing electrostatic attraction with BSP-A molecules (De Farias et al., 2022; Alves et al., 2019). At pH values above 9, BSP-A undergoes deprotonation, forming biphenolate anions. Simultaneously, the surfaces of Pyh and Pyh/AC also acquire negative charges, leading to electrostatic repulsion between the adsorbent and BSP-A, which reduces adsorption efficiency (Alves et al., 2019). This behavior highlights the importance of maintaining the optimal pH range for maximizing the decontamination performance of Pyh and Pyh/AC.

The influence of pH on the adsorption behavior of 4-CL shows that the adsorption efficiency using Pyh and Pyh/AC increases up to pH 6, reaching values of 63.4 mg/g for Pyh and 84.6 mg/g for Pyh/AC (Figure 6B). Beyond this, the adsorption remains stable at pH 7 but decreases significantly as the pH increases further, up to pH 10 (Figure 6B). In acidic conditions, the abundance of H^+^ ions leads to competitive adsorption on the carbon nanofiber surfaces, reducing the adsorption capacity of the target pollutant (4-CL) (Mao et al., 2022). The pKa values of 4-CL range from 4.7 to 9.4, and as the solution pH shifts from acidic to neutral or alkaline, 4-CL transitions from a neutral to a negatively charged ionic species. This shift enhances adsorption efficiency under neutral conditions (Hadi et al., 2021). However, when the pH exceeds the pKa of 4-CL, the molecule becomes ionized, acquiring a negative charge. This negative charge creates electrostatic repulsion with the negatively charged adsorbent surface, leading to a sharp decline in adsorption capacity under highly alkaline conditions (Gao et al., 2020a).

#### 3.2.2 Contact time

**FIGURE 7:**
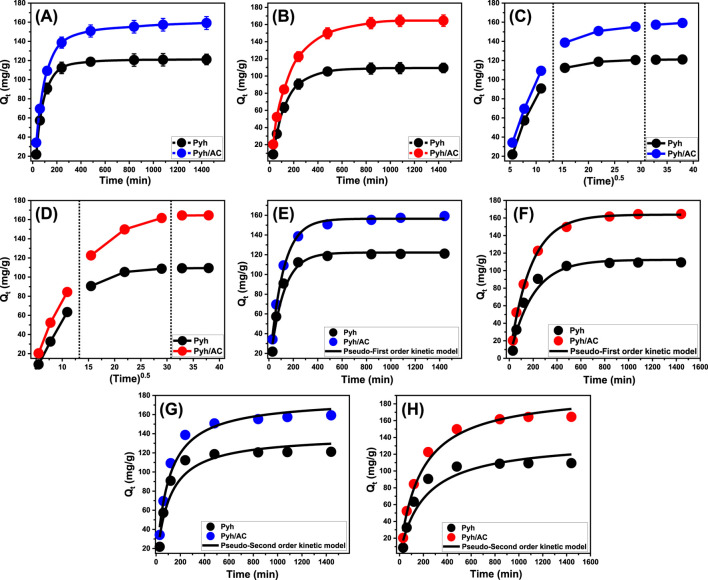
Shows the experimental influence of contact time on the adsorption properties of Pyh and Pyh/AC [BSP-A **(A)** and 4-CL **(B)**], the intra-particle diffusion curves for the adsorption processes [BSP-A **(C)** and 4-CL **(D)**], fitting of the adsorption results with Pseudo-First order kinetic model [BSP-A **(E)** and 4-CL **(F)**], and fitting of the adsorption results with Pseudo-Second order kinetic model [BSP-A **(G)** and 4-CL **(H)**].

An experiment was conducted to evaluate the adsorption properties of Pyh and Pyh/AC and their performances in removing BSP-A and 4-CL over a time range of 30–1,440 min. Key parameters, including the starting concentration (100 mg/L), temperature (293 K), solution volume (100 mL), pH (8 for BSP and 6 for 4-CL), and adsorbent dosage (0.2 g/L), were held constant while the effect of varying contact times was studied. The results demonstrated a significant improvement in the performances of both Pyh and Pyh/AC to adsorb BSP-A and 4-CL, as indicated by the amounts of molecules removed and their corresponding adsorption rates (Figures 7A, B). It was observed that the adsorption efficiencies of BSP-A increased substantially during 480 min (Pyh) and 840 min (Pyh/AC), with notable uptake improvements (Figure 7A). These time frames extend for 840 min (Pyh) and 1,080 min (Pyh/AC) during the uptake of 4-CL molecules (Figure 7B). However, beyond these time intervals or equilibrium points, no significant changes were detected in the removal rates or adsorption capacities of BSP-A and 4-CL, either by Pyh or by Pyh/AC. The findings suggest that Pyh and its composite with activated carbon (Pyh/AC) reached their maximum adsorption equilibrium after the previously mentioned time intervals. The determined equilibrium adsorption capacities of BSP-A and 4-CL using Pyh were 121.1 mg/g and 109 mg/g, respectively. The detected capacities using Pyh/AC were 159.2 mg/g for BSP-A and 164.4 mg/g for 4-CL. In the initial stages of the experiments, the adsorption and binding of BSP-A and 4-CL molecules increased at strong rates and in significant quantities, attributed to the availability of abundant active and vacant sites on the interfaces of Pyh and Pyh/AC (El-Sherbeeny et al., 2021). However, as the contact time increased, the number of unoccupied sites decreased due to prolonged molecule adsorption, which exhausted the available adsorption sites. This led to a decline in the uptake rates of BSP-A and 4-CL after the optimal contact period. At equilibrium, the Pyh and Pyh/AC adsorbents showed negligible improvements in adsorption performance, indicating that all functional sites were fully occupied. As a result, no further adsorption of BSP-A and 4-CL ions occurred, marking a stable state for the Pyh and Pyh/AC adsorbents (Abdel Salam et al., 2022).

#### 3.2.3 Kinetic studies

##### 3.2.3.1 Intra-particle diffusion behavior

The study of intra-particle diffusion characteristics during the adsorption processes using Pyh and Pyh/AC provides a detailed understanding of the mechanisms and binding interactions of BSP-A and 4-CL molecules. As shown in Figures 7C, D, the adsorption curves are divided into three distinct segments, each characterized by a different slope. These variations in slope reflect multiple adsorption stages occurring simultaneously alongside the diffusion behavior of BSP-A and 4-CL molecules (El Qada, 2020). The analysis suggests that the adsorption process involves three primary phases. (1) External adsorption (surface interactions): This phase involves the interaction of free surface sites within the Pyh and Pyh/AC frameworks with the phenol molecules. During this stage, BSP-A and 4-CL molecules are primarily bound to the outer surface of the Pyh and Pyh/AC nanoparticles through external adsorption mechanisms. (2) Internal adsorption (layered adsorption and diffusion): this phase occurs as the process progresses, involving layered uptake mechanisms where the molecules penetrate deeper into the nanoparticle structure and bind to the interior sites. The diffusion of BSP-A and 4-CL molecules towards the surface and internal layers of the adsorbents influences this stage. (3) Equilibrium or saturation phase: In this final stage, the adsorption process reaches a stable state as all available binding sites on the adsorbents are occupied, indicating the equilibrium or saturation point of the systems (Lin et al., 2021).

The initial findings reveal that the external uptake mechanism plays a dominant role during the early stages of the adsorption process. This phase is crucial as it governs the binding of BSP-A and 4-CL molecules to the surfaces of Pyh and Pyh/AC particles. The efficiency of this phase depends largely on the availability of active receptor sites on the surfaces of the nanoparticles. External adsorption is also responsible for regulating the majority of the remediation process (Figures 7C, D). As the adsorption process continues and the external surface sites become saturated, the internal or layered adsorption phase begins. This phase introduces a secondary removal pathway, where the ions diffuse into the internal structures of Pyh and Pyh/AC particles. The transition to this stage is observed when the external sites are fully occupied. In addition to layered adsorption, the diffusion behavior of both BSP-A and 4-CL molecules also plays a key role in this phase, further influencing the adsorption process (Figures 7C, D) (Lin et al., 2021; Neolaka et al., 2018).In the final stage, equilibrium is reached, and the adsorption process stabilizes as all effective binding sites, both external and internal, are occupied. At this point, BSP-A and 4-CL molecules are no longer removed through the mechanisms observed in earlier phases. Instead, molecular and interionic attraction forces come into play, facilitating the final removal of these ions. This stage confirms the completion of the binding process, with BSP-A and 4-CL molecules fully occupying the available sites on the Pyh and Pyh/AC particles, marking the saturation states of the materials (Salam et al., 2020; Sayed et al., 2022). This detailed progression through external adsorption, internal or layered retention, and equilibrium-affecting mechanisms highlights the complex yet systematic adsorption behavior of BSP-A and 4-CL molecules using Pyh and Pyh/AC. The process effectively utilizes the adsorbent’s surface and internal sites, ensuring high efficiencies in ions removal.

##### 3.2.3.2 Kinetic modeling

Understanding the adsorption kinetics of BSP-A and 4-CL onto Pyh and Pyh/AC is essential for determining the rate-controlling mechanisms and evaluating the interplay between physical and chemical interactions during the adsorption process. The kinetics of adsorption are primarily governed by mass transfer phenomena, including external diffusion, pore diffusion, and surface interactions, as well as chemical processes such as hydrogen bonding and complex formation (Neolaka et al., 2023). To analyze the dynamic behavior of adsorption, two commonly used kinetic models—the pseudo-first-order (P.F.) and pseudo-second-order (P.S.) models—were systematically applied to the experimental data. The pseudo-first-order (P.F.) model, derived from the Lagergren equation, describes adsorption processes where the rate of adsorption is proportional to the number of available adsorption sites (Neolaka et al., 2023). It assumes a physisorption-dominated mechanism, where weak van der Waals interactions and electrostatic forces primarily facilitate pollutant adsorption onto the adsorbent surface (Sherlala et al., 2019). On the other hand, the pseudo-second-order (P.S.) model, formulated by Ho and McKay, assumes that adsorption occurs through chemisorption, involving electron exchange or covalent bonding between the adsorbate and the functional groups on the adsorbent surface (Huang et al., 2018).

Non-linear regression fitting of the kinetic data to both models was performed to determine the best fit, using correlation coefficients (*R* ^2^) and chi-squared (χ^2^) values as indicators of model accuracy. The analysis (Table 1; Figures 7E–H) revealed that the pseudo-first-order model exhibited superior correlation with the experimental results, with *R* ^2^ values exceeding 0.97 for BSP-A and 4-CL adsorption on both Pyh and Pyh/AC. This strong agreement suggests that physical adsorption mechanisms—primarily van der Waals forces and electrostatic attractions—dominate the adsorption process. Furthermore, the theoretical maximum adsorption capacities obtained from the P.F. model (122.2 mg/g for BSP-A and 112.3 mg/g for 4-CL using Pyh, and 160.5 mg/g for BSP-A and 167.8 mg/g for 4-CL using Pyh/AC) closely matched the experimentally observed capacities, further validating the model’s applicability. This supports the hypothesis that physisorption is the primary mechanism driving the adsorption process (Sherlala et al., 2019; Huang et al., 2018).

**TABLE 1:** The mathematical parameters of the studied kinetic models.

Material	Model	Parameters	BSP-A	4-CL
Pyh	Pseudo-First-order	K_1_ (min^−1^)	0.0092	0.0052
		Qe _(Cal)_ (mg/g)	122.2	112.3
		*R* ^2^	0.98	0.97
		X^2^	0.72	1.69
	Pseudo-Second-order	k_2_ (g mg^-1^ min^−1^)	7.24 X 10^–5^	3.49 X 10^–5^
		Qe _(Cal)_ (mg/g)	138.5	137.1
		*R* ^2^	0.95	0.95
		X^2^	1.79	2.79
Pyh/AC	Pseudo-First-order	K_1_ (min^-1^)	0.0093	0.0056
		Qe _(Cal)_ (mg/g)	160.5	167.8
		*R* ^2^	0.99	0.99
		X^2^	0.14	0.35
	Pseudo-Second-order	k_2_ (g mg^-1^ min^−1^)	5.93 X 10^–5^	2.88 X 10^–5^
		Qe _(Cal)_ (mg/g)	176.7	195.4
		*R* ^2^	0.98	0.98
		X^2^	0.77	0.91

Although the pseudo-first-order model provided a better fit, the pseudo-second-order model also demonstrated reasonable agreement with the data. This suggests that while chemisorption is not the dominant mechanism, secondary interactions such as hydrogen bonding and complex formation contribute to the adsorption process. The observed slight deviation from the P.F. model in some cases could be attributed to the potential role of functional groups on Pyh and Pyh/AC in forming weak chemical interactions with BSP-A and 4-CL molecules (Salam et al., 2020; Sherlala et al., 2019).

The study also highlighted the possibility of successive adsorption layers, particularly at high pollutant concentrations. Initially, a single layer of BSP-A and 4-CL molecules is physically adsorbed onto the active sites of Pyh and Pyh/AC, driven by van der Waals forces and electrostatic attractions. As adsorption progresses, additional pollutant molecules may be retained through secondary interactions, such as dipole-dipole forces or weak hydrogen bonding, leading to an apparent increase in adsorption capacity beyond monolayer coverage (Jasper et al., 2020). This is consistent with the observed steric properties, which indicate that each active site on Pyh/AC can accommodate multiple molecules. The difference in adsorption behavior between BSP-A and 4-CL can be attributed to molecular structure and functional group interactions. The lower adsorption energy of 4-CL suggests a weaker interaction with the adsorbent surface, which may lead to greater reliance on physisorption. In contrast, the higher adsorption energy for BSP-A indicates stronger intermolecular forces, potentially leading to partial chemisorption effects in some cases (Mobarak et al., 2021).

These findings have significant practical implications for wastewater treatment applications. Since physical adsorption is reversible, the Pyh/AC composite can be easily regenerated and reused, making it a cost-effective and sustainable adsorbent. The dominance of van der Waals and electrostatic interactions ensures rapid adsorption kinetics, making Pyh/AC suitable for high-throughput water purification systems.

#### 3.2.4 Starting concentration

**FIGURE 8:**
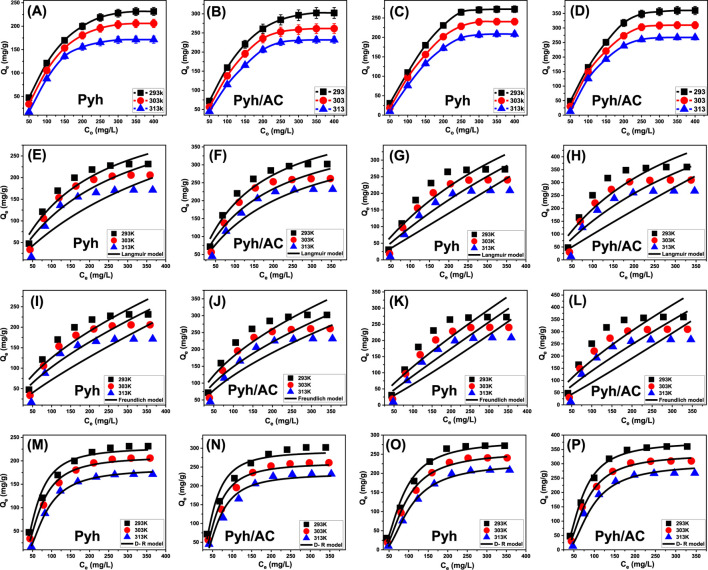
Shows the experimental influence of starting pollutants concentrations on the adsorption properties of Pyh and Pyh/AC [BSP-A **(A, B)** and 4-CL **(C, D)**], fitting of the experimental results with Langmuir isotherm [BSP-A **(E, F)** and 4-CL **(G, H)**], fitting of the results with Freundlich isotherm [BSP-A **(I, J)** and 4-CL **(K, L)**], and fitting of the results with D-R isotherm [BSP-A **(M, N)** and 4-CL **(O, P)**].

This study examined how initial concentrations of BSP-A and 4-CL influence their maximum adsorption capacities on Pyh and Pyh/AC, as well as the corresponding equilibrium conditions, within a concentration range of 50–450 mg/L. The experiments were conducted under controlled parameters, including a solution volume of 100 mL, a contact time of 24 h, an adsorbent dosage of 0.2 g/L, pH levels (8 for BSP-A and 6 for 4-CL), and temperatures varying from 293 K to 313 K. The results demonstrated a strong relationship between increasing concentrations of BSP-A (Figures 8A, B) and 4-CL (Figures 8C, D) and their adsorption capacities on Pyh and Pyh/AC. Higher initial concentrations of BSP-A and 4-CL increased their diffusion rates, driving forces, and mobility within the solution. These factors facilitated the interaction of the molecules with a larger number of free and reactive binding sites on the adsorbent surfaces. Consequently, the adsorption efficiency improved as the concentration of BSP-A and 4-CL increased systematically, but only up to a specific threshold (Ahmed et al., 2024b). Beyond this point, further increases in the concentrations did not significantly enhance adsorption efficiency, indicating that the active sites on the adsorbent surface had reached saturation. Identifying the equilibrium point is therefore essential for determining the maximum adsorption capacity of Pyh and Pyh/AC. The study determined that the maximum adsorption capacities of BSP-A on Pyh were 231.4 mg/g at 293 K, 205.8 mg/g at 303 K, and 171.3 mg/g at 313 K (Figure 8A). For 4-CL, the capacities on Pyh were 272.4 mg/g at 293 K, 240.5 mg/g at 303 K, and 208.5 mg/g at 313 K (Figure 8C). When Pyh/AC was used, the adsorption capacity for BSP-A were 302.2 mg/g at 293 K, 261.5 mg/g at 303 K, and 231.7 mg/g at 313 K (Figure 8B). However, for 4-CL, Pyh/AC demonstrated significantly higher adsorption capacities, reaching 360.3 mg/g at 293 K, 309.7 mg/g at 303 K, and 267.4 mg/g at 313 K (Figure 8D). The decline in adsorption capacity observed at higher temperatures for both BSP-A and 4-CL suggests that the adsorption process is exothermic. Additionally, the enhanced performance of Pyh/AC compared to Pyh highlights the beneficial role of the activated carbon substrate. Activated carbon increases the surface area, enhances the number of active functional groups, and improves the reactivity of the adsorption interface. Furthermore, its porous structure offers additional opportunities for interactions with internal surfaces, providing more binding sites. These attributes make Pyh/AC a more effective adsorbent for BSP-A and 4-CL, as it combines the strengths of both Pyh and activated carbon to deliver enhanced adsorption performance.

#### 3.2.5 Classic isotherm models

The study of equilibrium adsorption is critical for determining the efficiency, capacity, and selectivity of an adsorbent toward target pollutants in aqueous solutions. Equilibrium models describe the distribution of adsorbates between the solid phase (adsorbent) and liquid phase (solution) at a given temperature, providing insights into key parameters such as adsorption capacity, surface homogeneity, and the nature of adsorbate-adsorbent interactions (Sherlala et al., 2019; Dawodu et al., 2012). To characterize the adsorption behavior of BSP-A and 4-CL on Pyh and Pyh/AC, three widely recognized isotherm models were applied: Langmuir (Figures 8E, H), Freundlich (Figures 8I–L), and Dubinin-Radushkevich (D-R) (D-R (Figures 8M–P). The Langmuir isotherm model is based on the assumption that adsorption occurs in a monolayer fashion on a surface with homogeneous active sites, where each site can accommodate only one adsorbate molecule (Huang et al., 2018; Dawodu et al., 2012). The Freundlich model describes adsorption on heterogeneous surfaces where multiple adsorption sites of varying affinity are available (Dawodu et al., 2012). Non-linear regression techniques were employed to compare the predicted equilibrium parameters of these models with the experimentally observed adsorption behaviors of BSP-A and 4-CL. Key performance indicators, including the correlation coefficient (*R* ^2^) and Chi-squared (χ^2^) values, were analyzed to determine the accuracy and applicability of the models.

The experimental results indicate a strong correlation between the adsorption of BSP-A and 4-CL on Pyh and Pyh/AC with the Langmuir model (Table 2; Figures 8E, H). The high correlation coefficients (*R* ^2^ > 0.90) suggest that adsorption occurs predominantly as a monolayer on the surface, supporting the assumption that adsorption sites are evenly distributed across the Pyh and Pyh/AC frameworks (Dawodu et al., 2012). Additionally, the dimensionless separation factor (R_L_), which determines the favorability of adsorption, was found to be less than 1 for both BSP-A and 4-CL, indicating a favorable adsorption process (Table 2) (El Qada, 2020). The theoretical maximum adsorption capacities (Q_max_) derived from Langmuir fitting further validate the experimental findings: For Pyh, Q_max_ values for BSP-A were 294.6 mg/g (293 K), 225.5 mg/g (303 K), and 197.9 mg/g (313 K), while for 4-CL, Q_max_ values were 295.3 mg/g (293 K), 263.6 mg/g (303 K), and 228.3 mg/g (313 K) (Table 2). For Pyh/AC, the adsorption capacities were significantly enhanced, with BSP-A reaching 374.9 mg/g (293 K), 306.5 mg/g (303 K), and 260.2 mg/g (313 K) and 4-CL showing adsorption capacities of 420.6 mg/g (293 K), 379.6 mg/g (303 K), and 300.2 mg/g (313 K) (Table 2). These results confirm that Pyh/AC provides a higher adsorption capacity than Pyh alone due to increased porosity, improved functional site distribution, and enhanced molecular interactions. The agreement with the Langmuir model suggests that surface saturation is achieved at equilibrium, limiting further adsorption beyond monolayer coverage (Huang et al., 2018; Dawodu et al., 2012).

**TABLE 2:** The mathematical characteristics of the traditional isotherm models under consideration.

Materials	Models	Parameters	BSP-A			4-CL		
			293 K	303 K	313 K	293 K	303 K	313 K
Pyh	Langmuir	*Q*_max_ (mg/g)	294.6	225.5	197.9	295.3	263.6	228.3
		*b*(L/mg)	0.0052	0.0038	0.002	0.002	9.2 x 10^−4^	2.3 x 10^−7^
		*RL*	0.80–0.33	0.84–0.39	0.91–0.57	0.91–0.57	0.95–0.73	0.999
		*R* ^*2*^	0.94	0.92	0.90	0.90	0.89	0.88
		*X* ^*2*^	3.2	4.3	5.44	4.3	4.8	5.6
	Freundlich	*1/n*	0.60	0.67	0.83	0.81	0.88	0.92
		*k*_*F*_ (mg/g)	7.8	4.5	1.5	2.6	1.9	1.2
		*R* ^*2*^	0.90	0.87	0.83	0.87	0.86	0.85
		*X* ^*2*^	6.2	8.2	9.8	7.4	7.8	8.3
	D-R	*β* (mol^2^/kJ^2^)	0.0067	0.0069	0.0072	0.0051	0.0058	0.0066
		*Q*_*m*_ (mg/g)	238.7	209.1	183.4	285.2	255.8	217.1
		*R* ^*2*^	0.98	0.99	0.99	0.99	0.99	0.99
		*X* ^*2*^	1.00	0.36	0.08	1.16	0.30	0.15
		*E* (kJ/mol)	8.63	8.51	8.33	9.9	9.28	8.70
Pyh/AC	Langmuir	*Q*_max_ (mg/g)	374.9	306.5	260.2	420.6	379.6	300.2
		*b* (L/mg)	0.0071	0.006	0.0045	0.0032	0.0021	2.5 × 10^−4^
		*RL*	0.74–0.26	0.77–0.29	0.81–0.35	0.86–0.43	0.91–0.55	0.98–0.90
		*R* ^*2*^	0.96	0.96	0.93	0.90	0.88	0.85
		*X* ^*2*^	2.4	3.8	3.8	4.1	5.3	6.2
	Freundlich	*1/n*	0.54	0.57	0.63	0.73	0.83	0.93
		*k*_*F*_ (mg/g)	14.8	10.5	6.6	6.1	3.01	2.68
		*R* ^*2*^	0.90	0.87	0.88	0.84	0.82	0.79
		*X* ^*2*^	6.1	7.6	7.1	7.7	8.2	8.7
	D-R	*β* (mol^2^/kJ^2^)	0.0057	0.0062	0.0071	0.0046	0.0054	0.0063
		*Q*_*m*_ (mg/g)	309.6	264.0	232.2	366.9	322.9	289.4
		*R* ^*2*^	0.97	0.98	0.97	0.99	0.99712	0.99
		*X* ^*2*^	2.1	1.2	1.45	0.23	0.41	1.95
		*E* (kJ/mol)	9.36	8.98	8.39	10.42	9.62	8.91

While the Langmuir model provided a better fit, the Freundlich model also displayed reasonable correlation (Figures 8I–L). The values of 1/n ranged from 0.5 to 0.9, suggesting moderately heterogeneous adsorption behavior. This implies that some adsorption sites exhibit higher affinity than others, possibly due to variations in functional group distribution or surface roughness (Dawodu et al., 2012). However, the relatively lower correlation coefficients for the Freundlich model compared to the Langmuir model indicate that multilayer adsorption is not the dominant mechanism in this system. Instead, adsorption primarily occurs through monolayer interactions, with minor contributions from multilayer formation at high concentrations (Dawodu et al., 2012).

The equilibrium properties from the Dubinin-Radushkevich (D-R) isotherm model provide insights into the energetic variations in the adsorption processes of BSP-A and 4-CL using Pyh and Pyh/AC nanostructures. Unlike other models, the D-R model evaluates adsorption energy (E) distribution, helping to identify whether the mechanism is physical, chemical, or a combination (Kusuma et al., 2024). Adsorption mechanisms are categorized based on E values: (A) Below 8 kJ/mol: strong physical adsorption (e.g., van der Waals forces), (B) 8–16 kJ/mol: combined physical and chemical adsorption (e.g., hydrogen bonding), and (C) above 16 kJ/mol: strong chemical adsorption (e.g., covalent bonding) (Kusuma et al., 2024; Ahmed et al., 2024a). The calculated E values for BSP-A and 4-CL ranged between 8 and 16 kJ/mol (Table 2), suggesting a combination of physical and chemical interactions. This indicates that while physisorption mechanisms (e.g., van der Waals forces, π-π stacking, electrostatic attraction) play a dominant role, weak chemical interactions such as hydrogen bonding contribute to adsorption stability (Kusuma et al., 2024; Ahmed et al., 2024a).

The high surface area and porosity of Pyh/AC facilitate physisorption, while nitrogen-containing functional groups in polypyrrole contribute to hydrogen bonding interactions, reinforcing overall adsorption efficiency. This dual-mode adsorption mechanism allows for strong pollutant retention while maintaining reversible desorption properties, which is essential for regeneration and recyclability. The strong agreement between theoretical predictions and experimental findings validates the adsorption behavior of Pyh/AC, demonstrating its potential as a high-capacity, efficient, and reusable adsorbent for BSP-A and 4-CL removal.

#### 3.2.6 Advanced isotherm models

A statistical physics model has been utilized to describe the equilibrium aspects of adsorption tendencies, providing an in-depth analysis of the distinct characteristics of these reactions. This approach focuses on the interactions between water-soluble pollutants and the primary reactive chemical groups that act as receptors on the surface of adsorbents. Using advanced mathematical simulations, the study captures both steric and energetic factors, offering a comprehensive understanding of the adsorption mechanisms. The steric parameters analyzed include Nm (the total number of occupied adsorption sites on the surfaces of Pyh and Pyh/AC), the number of molecules anchored per receptor (n), and the maximum adsorption capacities (Q_sat_) of BSP-A and 4-CL at full saturation when adsorbed on Pyh and Pyh/AC. These steric factors are crucial because they provide insights into the structural and spatial limitations that influence adsorption efficiency. Additionally, the energetic and thermodynamic parameters studied include internal energy (E_int_), entropy (Sa), adsorption energy (ΔE), and free enthalpy (G). These factors provide a deeper understanding of the driving forces and spontaneity of the adsorption process. The energy parameters determine whether adsorption is favorable, while entropy changes highlight the disorder introduced during adsorption.

**FIGURE 9:**
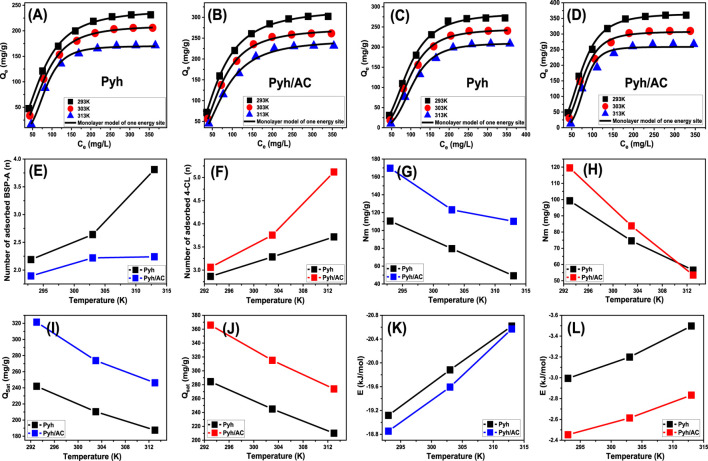
Fitting of the experimental results with advanced monolayer model of one energy site [BSP-A **(A B)** and 4-CL **(C, D)**], changes in the number of adsorbed molecules per each active site [BSP-A **(E)** and 4-CL **(F)**], changes in the occupies active sites density during the adsorption processes [BSP-A **(G)** and 4-CL **(H)**], changes in the saturation uptake capacities of Pyh and Pyh/AC [BSP-A **(I)** and 4-CL **(J)**], and changes in the adsorption energies of Pyh and Pyh/AC [BSP-A **(K)** and 4-CL **(L)**].

To validate the proposed models, a non-linear regression analysis was conducted using assumptions of different presented models (Supplementary Table S1). The Levenberg-Marquardt iterative method, combined with multivariable non-linear regression, was applied to achieve precise fitting of the experimental data. The adsorption behavior of BSP-A and 4-CL on Pyh and Pyh/AC was evaluated, and the results demonstrated strong fitting accuracy with the single active-site monolayer model, which was employed to analyze the adsorption process. This model assumes that adsorption occurs at specific, individual active sites without interaction between adsorbed molecules. The fitting results confirmed the reliability of this approach, as shown in Figures 9A–D and Table 3, which also provide detailed calculated parameters for the model.

**TABLE 3:** The mathematical parameters obtained for the assessed Monolayer model of one energy site.

Materials	Parameters	BSP-A			4-CL		
		293 K	303 K	313 K	293 K	303 K	313 K
Pyh	*R* ^*2*^	0.999	0.999	0.999	0.999	0.998	0.998
	*X* ^*2*^	0.03	0.04	0.08	0.12	0.31	0.35
	*n*	2.19	2.64	3.81	2.86	3.28	3.72
	*Nm* (mg/g)	110.5	79.6	49.2	99.3	74.6	56.5
	*Q*_*sat*_ (mg/g)	241.8	210.4	187.5	284.3	244.9	210.1
	*C1/2* (mg/L)	76.8	80.3	82.8	92.3	96.1	103.5
	*ΔE* (kJ/mol)	−19.1	−19.8	−20.6	−2.9	−3.2	−3.5
Pyh/CA	*R* ^*2*^	0.994	0.999	0.998	0.998	0.994	0.992
	*X* ^*2*^	0.03	0.06	0.13	0.21	0.96	1.73
	*n*	1.89	2.22	2.24	3.06	3.76	5.12
	*Nm* (mg/g)	169.7	123.1	110.2	119.5	83.8	53.5
	*Q*_*sat*_ (mg/g)	321.4	273.8	246.3	365.8	315.1	273.9
	*C1/2* (mg/L)	68.9	71.6	81.3	73.9	76.2	80.2
	*ΔE* (kJ/mol)	−18.8	−19.6	−20.6	−2.45	−2.6	−2.8

##### 3.2.6.1 Steric properties

###### 3.2.6.1.1 Number of adsorbed molecules per site (n)

The theoretical analysis of the *n* value offers detailed insights into the spatial arrangement of BSP-A and 4-CL molecules adsorbed onto the surfaces of Pyh and Pyh/AC, encompassing both horizontal and vertical orientations. These observations are fundamental for comprehending the adsorption mechanisms, which involve multi-molecular or multi-docking interactions. *n* values below 1 indicate a horizontal configuration of adsorbed molecules, suggesting limited molecular layering at a single adsorption site and a controlling effect for the multi-docking mechanism. This suggests weak intermolecular forces, such as van der Waals interactions, dominate the adsorption process. Conversely, *n* values exceeding 1 signify vertical or non-parallel alignments or multi-layered configurations, where multiple molecules occupy a single site through multi-ionic interactions. This might occur due to stronger interactions between the molecules and the adsorbent’s functional groups. Such behaviors reflect the ability of individual sites to host several molecules, effectively enhancing the adsorption process (Sayed et al., 2022; Mobarak et al., 2021).

For BSP-A adsorption on Pyh, the *n* values ranged between 2.19 and 3.81, whereas for 4-CL, the values were between 2.86 and 3.73 (Figures 9E, F; Table. 3). In contrast, adsorption on the Pyh/AC composite produced slightly lower *n* values for BSP-A, ranging from 1.89 to 2.24, but higher values for 4-CL, ranging from 3.06 to 5.12 (Figures 9E, F; Table. 3). These results confirm that multiple BSP-A or 4-CL molecules can occupy a single site, indicating a prominent role of multi-molecular interactions in the adsorption process. These compounds, as adsorbed molecules according to the estimated values, display vertical and non-parallel orientation on the surfaces of both Pyh and the Pyh/AC composite. The adsorption capacity of individual binding sites further reveals a difference between the two adsorbents. For BSP-A, Pyh accommodates up to 4 molecules per site, whereas Pyh/AC supports a slightly lower capacity of 3 molecules. In contrast, Pyh/AC exhibits superior capacity for 4-CL, accommodating up to 6 molecules per site, compared to only 4 molecules on Pyh. This disparity highlights the significant impact of the hybridization process on the reactivity of the interactive interface and the aggregation tendencies of BSP-A and 4-CL molecules during their interaction with the existing functional groups as reactive uptake centers. Furthermore, the *n* values for both BSP-A and 4-CL increase with rising temperature, indicating enhanced aggregation tendencies and intensified molecular collisions with the adsorbent surfaces (Figures 9E, F; Table. 3) (Mobarak et al., 2021; Dhaouadi et al., 2021). This also demonstrates the impact of the thermal activation process that could transpire prior to the adsorption of BSP-A and 4-CL (Mobarak et al., 2021; Dhaouadi et al., 2021).

###### 3.2.6.1.2 Density of the active sites (*Nm*)

Quantitative assessment of the functional uptake sites for BSP-A and 4-CL (Figures 9G, H) provides valuable estimates of the total number of adsorption sites occupied (*Nm*) on the surfaces of Pyh and Pyh/AC composite. For Pyh, the *Nm* values during BSP-A adsorption were measured as 110.5 mg/g at 293 K, 79.6 mg/g at 303 K, and 49.2 mg/g at 313 K (Figure 9G). Similarly, the *Nm* values for 4-CL adsorption on Pyh were 99.3 mg/g at 293 K, 74.6 mg/g at 303 K, and 56.5 mg/g at 313 K (Figure 9H). In comparison, the Pyh/AC composite exhibited significantly higher values, with BSP-A adsorption reaching 169.7 mg/g at 293 K, 123.1 mg/g at 303 K, and 110.2 mg/g at 313 K (Figure 9G). For 4-CL, the corresponding *Nm* values on Pyh/AC were 119.5 mg/g at 293 K, 83.8 mg/g at 303 K, and 53.5 mg/g at 313 K (Figure 9H). These findings demonstrate that the carbonization and activation of rice husk, followed by the incorporation of Pyh, substantially enhance the number of functional adsorption sites. This improvement can be attributed to the activation process, which increases the porosity of the rice husk-derived activated carbon, thereby enlarging the surface area and expanding the contact interface. The integration of Pyh further enriches the hybrid structure by providing additional active sites, contributing to the superior adsorption capacity of the Pyh/AC composite compared to standalone Pyh. The results also reveal a temperature-dependent variation in the number of occupied adsorption sites during the uptake of BSP-A and 4-CL (Figures 9G, H). A decline in *Nm* values with increasing temperature suggests a negative influence of elevated thermal conditions on the activity of existing reactive adsorption sites, causing sometimes deactivation of them. Also, this behavior may be explained by reduced contact time required for the effective anchoring of the soluble molecules into the existing sites by decreasing the viscosity of the solutions and in turn increasing their diffusion rates. Moreover, higher temperatures promote desorption by lowering the saturation limits in solution, leading to the release of adsorbed BSP-A and 4-CL molecules from the surfaces of Pyh and Pyh/AC (Sellaoui et al., 2020; Ahmed et al., 2024c). Additionally, the observed reduction in the number of occupied sites aligns with the trends reported for the *n* parameter, which increases with temperature. The rise in *n* values reflects a higher aggregation tendency of adsorbed molecules, which reduces the overall occupation of functional uptake sites.

###### 3.2.6.1.3 Adsorption capacity at the saturation state of (Q_sat_)

The fully saturated adsorption capacities (*Q*_*sat*_) of Pyh and Pyh/AC composite highlight their efficient uptake performance and stability in sequestering BSP-A and 4-CL molecules (Figures 9I, J. The two primary determinants of *Q*_*sat*_ are the density of occupied sites (*Q*_*sat*_) and the number of molecules occupying each site (*n*). For Pyh, the saturation adsorption capacities for BSP-A were recorded as 241.8 mg/g at 293 K, 201.4 mg/g at 303 K, and 187.5 mg/g at 313 K (Figure 9I). The corresponding values for 4-CL adsorption were 284.3 mg/g at 293 K, 244.9 mg/g at 303 K, and 210.1 mg/g at 313 K (Figure 9J). In comparison, the Pyh/AC composite demonstrated better adsorption capacities for BSP-A than Pyh, with values of 321.4 mg/g at 293 K, 273.8 mg/g at 303 K, and 246.3 mg/g at 313 K (Figure 9I). For 4-CL, the corresponding *Q*_*sat*_ values for Pyh/AC were 365.8 mg/g at 293 K, 315.1 mg/g at 303 K, and 273.9 mg/g at 313 K (Figure 9J). The observed trends suggest that the adsorption of BSP-A and 4-CL is exothermic in nature, as indicated by the inverse relationship between temperature and the marked saturation adsorption capacities (Figures 9I, J). These findings are consistent with prior studies, which demonstrated reduced adsorption capacity in thermally activated systems due to desorption effects and diminished binding stability in addition to the weakening of adsorbate-adsorbent interactions (Sayed et al., 2022; Sellaoui et al., 2020; Ahmed et al., 2024c). Furthermore, the temperature dependence of *Q*_*sat*_ mirrors the behavior of *Nm* rather than *n*, indicating that the total density of available adsorption sites plays a more significant role in determining adsorption efficiency than the multi-molecular occupancy of individual binding sites. Overall, the findings emphasize the enhanced performance of the Pyh/AC composite, which can be attributed to the increased surface area, enhanced porosity, and the greater number of functional active sites resulting from the hybridization process. This improved structural configuration allows for more efficient adsorption, making Pyh/AC a promising for the uptake of phenolic contaminants such as BSP-A and 4-CL.

##### 3.2.6.2 Energetic properties

###### 3.2.6.2.1 Adsorption energy and mechanism

The investigation into the energy variations (ΔE) associated with the binding of BSP-A and 4-CL molecules provides essential insights into the underlying mechanisms, whether they are chemical or physical in nature. Physical processes typically exhibit binding energies below 40 kJ/mol, while chemical processes are characterized by energy levels exceeding 80 kJ/mol. These binding energy thresholds serve as fundamental criteria for differentiating various physical interaction mechanisms. Physical processes include hydrogen bonding (<30 kJ/mol), dipole-dipole interactions (2–29 kJ/mol), coordination exchange (∼40 kJ/mol), van der Waals forces (4–10 kJ/mol), electrostatic attraction (2–50 kJ/mol), and hydrophobic interactions (∼5 kJ/mol). The adsorption energy (ΔE) for BSP-A and 4-CL molecules was estimated using Equation 5, which incorporates the solubility (S) of BSP-A and 4-CL, the gas constant (R = 0.008314 kJ/mol·K), the concentration of BSP-A and 4-CL at half-saturation conditions, and the system temperature (T) (Dhaouadi et al., 2020).

$$∆E=RT\;\ln\left(\frac{S}{C}\right)$$ (5)

The results revealed adsorption energies below 21 kJ/mol for BSP-A and below 4 kJ/mol for 4-CL in the Pyh and Pyh/AC systems (Figures 9K, L; Table 3). These values align with the energetic limits typically associated with physisorption. Furthermore, the negative ΔE values confirm that the interactions between BSP-A and 4-CL molecules and the Pyh/Pyh-AC frameworks are exothermic processes. Quantitative analysis indicates that hydrogen bonding, electrostatic interactions, and dipole-dipole interactions are the dominant physical processes contributing to the removal of BSP-A and 4-CL using Pyh and Pyh/AC. Hydrogen bonds typically occur between electronegative atoms (e.g., oxygen and nitrogen) and hydrogen atoms in hydroxyl (-OH), amine (-NH), or carboxyl (-COOH) functional groups. BSP-A and 4-CL contain polar functional groups, which facilitate their interaction with surface functionalities on Pyh and Pyh/AC, particularly through hydroxyl (-OH) and amine (-NH) sites in the polypyrrole hydrogel framework (Sellaoui et al., 2016; Foo and Hameed, 2009).

The presence of negatively charged functional groups in Pyh/AC (such as oxygenated species) enhances the electrostatic attraction between the adsorbent and the partially positive hydrogen atoms of BSP-A and 4-CL, leading to effective adsorption. The role of electrostatic interactions is consistent with the negative ΔE values, which indicate favorable and spontaneous interactions driven by energetic stability. On the other hand, both BSP-A and 4-CL molecules possess high dipole moments, allowing them to interact with the polar sites on the adsorbent surface, contributing to their overall physisorption behavior. The relatively higher adsorption energy of BSP-A (21 kJ/mol) compared to 4-CL (4 kJ/mol) suggests that dipole-dipole interactions play a greater role in BSP-A adsorption, likely due to its larger molecular structure and multiple polar functional groups. Regarding the impact of Van der Waals Forces, these weak intermolecular forces contribute to non-specific adsorption, particularly for 4-CL, which exhibits lower adsorption energy and fewer hydrogen bonding interactions compared to BSP-A. This suggests that van der Waals forces are the dominant mechanism for 4-CL adsorption, leading to weaker retention and easier desorption.

The differences in adsorption energy for BSP-A and 4-CL can be attributed to variations in molecular size, polarity, and functional group composition. BSP-A, with its higher adsorption energy (21 kJ/mol), demonstrates stronger interactions compared to 4-CL (4 kJ/mol). This discrepancy may result from greater hydrogen bonding potential or stronger dipole interactions for BSP-A due to its larger molecular structure and multiple polar functional groups. Conversely, the lower energy for 4-CL suggests weaker physical interactions, likely dominated by van der Waals forces or electrostatic attractions and minimal hydrogen bonding as structure contains one hydroxyl (-OH) and one chlorine (-Cl) group in addition to its smaller molecular size. The significantly lower adsorption energy of 4-CL suggests that desorption is more efficient, making Pyh/AC particularly useful for treating chlorinated phenolic contaminants that require easy regeneration. In contrast, the higher energy of BSP-A adsorption indicates stronger pollutant-adsorbent interactions, which could be beneficial for achieving high removal efficiencies in wastewater treatment applications.

###### 3.2.6.2.2 Thermodynamic functions

####### 3.2.6.2.2.1. Entropy

The entropy (*Sa*) characteristics associated with the adsorption of BSP-A and 4-CL molecules on Pyh and Pyh/AC provide clear insights into the ordered and disordered nature of the adsorbent surface interfaces under varying concentrations of BSP-A and 4-CL molecules and at different temperatures. These distinct *Sa* features can be evaluated through the results obtained from Equation 6, which relies on the previously determined values of *Nm* and *n*, as well as the expected concentration of BSP-A and 4-CL at the half-saturation states (*C1/2*) of Pyh and Pyh/AC:

$$\frac{S_{a}}{K_{B}}=Nm\left\{\ln\left(1+{\left(\frac{C}{C_{\frac{1}{2}}}\right)}^{n}\right)-n{\left(\frac{C}{C_{\frac{1}{2}}}\right)}^{n}\;\frac{\ln\left(\frac{C}{C_{\frac{1}{2}}}\right)}{1+{\left(\frac{C}{C_{\frac{1}{2}}}\right)}^{n\;}}\;\right\}$$ (6)

**FIGURE 10:**
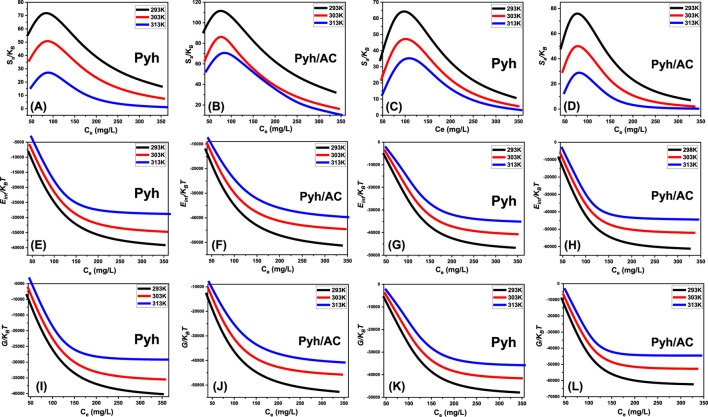
Shows the changes in the thermodynamic parameters during the uptake processes including entropy [BSP-A **(A, B)** and 4-CL **(C, D)**], internal energy [BSP-A **(E, F)** and 4-CL **(G, H)**], and free enthalpy [BSP-A **(I, J)** and 4-CL **(K, L)**].

Analysis of the resulting graphs indicates a significant decline in entropy (*Sa*) following the adsorption of BSP-A and 4-CL molecules onto Pyh and Pyh/AC surfaces, particularly at higher concentrations of these phenolic compounds (Figures 10A–D). This reduction highlights a substantial decrease in the disorder of Pyh and Pyh/AC surface interfaces as the concentration of BSP-A and 4-CL increases. The entropy analysis further reveals an improvement in the adsorption efficiency of BSP-A and 4-CL molecules onto the vacant binding sites of Pyh and Pyh/AC, even at relatively low concentrations of these compounds (Sellaoui et al., 2020; Dhaouadi et al., 2020).

The maximum entropy values for BSP-A adsorption on Pyh were observed at equilibrium concentrations of 75.8 mg/L (293 K), 78.9 mg/L (303 K), and 82.5 mg/L (313 K) (Figure 10A). For 4-CL adsorption onto Pyh, the corresponding equilibrium concentrations were 78.1 mg/L (293 K), 80.6 mg/L (303 K), and 84.8 mg/L (313 K) (Figure 10C). Similarly, for BSP-A adsorption onto Pyh/AC, the equilibrium concentrations were recorded as 68.2 mg/L at 293 K, 72.5 mg/L at 303 K, and 77 mg/L at 313 K (Figure 10B). The adsorption of 4-CL onto Pyh/AC exhibited comparable equilibrium concentrations: 67.1 mg/L at 293 K, 70.2 mg/L at 303 K, and 74.9 mg/L at 313 K (Figure 10D). These equilibrium concentrations align closely with those obtained at the half-saturation states of Pyh and Pyh/AC. Consequently, the binding of additional molecules is hindered due to the limited availability of residual uptake sites. The pronounced decreases in entropy signify a marked reduction in the number of available adsorption sites, accompanied by a notable restriction in the mobility and diffusion of BSP-A and 4-CL molecules on the adsorbent surfaces (Sellaoui et al., 2016).

####### 3.2.6.2.2.2. Internal energy and free enthalpy

This study investigated the internal energy (*E*_*int*_) associated with the removal of BSP-A and 4-CL molecules by Pyh and Pyh/AC, alongside the evaluation of free enthalpy (*G*), while considering variations in starting concentrations and the effects of system temperature. The analysis of *E*_*int*_ and *G* offers insights into the spontaneity, feasibility, and energetic changes associated with the removal process under varying conditions, particularly temperature. The analysis was conducted using Equations 7, 8, which incorporate the determined values of *Nm*​, *n*, and *C1/2*, as well as the translational partition function (Zv) (Dhaouadi et al., 2021).

$$\frac{E_{\mathrm{int}}}{K_{B}T\;}=n\;N_{m}\left[\left(\;\frac{{\left(\frac{C}{C_{1/2}}\right)}^{n}\;\ln\left(\frac{C}{Z_{v}}\right)}{1+{\left(\frac{C}{C_{1/2}}\right)}^{n}}\right)-\left(\;\frac{n\ln\left(\frac{C}{C_{1/2}}\right)\;{\left(\frac{C}{C_{1/2}}\right)}^{n}}{1+{\left(\frac{C}{C_{1/2}}\right)}^{n}}\right)\right]$$ (7)

$$\frac{G}{K_{B}T\;}=n\;N_{m}\frac{\ln\left(\frac{C}{Z_{v}}\right)}{1+{\left(\frac{C_{1/2}}{C}\right)}^{n}}$$ (8)

The observed changes in *E*_*int*_ during the removal of BSP-A and 4-CL molecules by Pyh and Pyh/AC were consistently negative. Notably, a significant reduction in *E*_*int*_ was recorded as the temperature increased from 293 K to 313 K (Figures 10E–H). These findings confirm the exothermic, spontaneous, and thermodynamically favorable nature of the adsorption processes involving Pyh and Pyh/AC. Furthermore, the observed energy reduction at elevated temperatures may indicate a reduction in the degree of molecular mobility or a transition to a more energetically stable configuration. Furthermore, the enthalpy results revealed trends comparable to those observed for internal energy, reinforcing the consistency of the thermodynamic behavior. The free enthalpy (*G*) demonstrated a reversible dependence on the adsorption temperature (Figures 10I–L), indicating a reduction in thermodynamic feasibility while reaffirming the exothermic and spontaneous characteristics of the adsorption processes for BSP-A and 4-CL molecules using Pyh and Pyh/AC.

#### 3.2.7 Recyclability

The ability to regenerate and reuse an adsorbent without significant loss of efficiency is a crucial factor for its practical application, economic feasibility, and environmental sustainability in wastewater treatment. The Pyh/AC composite demonstrated strong recyclability, maintaining high adsorption capacities for bisphenol-A (BSP-A) and 4-chlorophenol (4-CL) over five consecutive adsorption-desorption cycles. The regeneration process was performed by washing the exhausted Pyh/AC particles with distilled water, followed by drying at 50°C for 8 h to remove residual moisture and prepare the material for reuse.

Experimental results confirmed that Pyh/AC retains over 80% of its initial adsorption capacity after five cycles, with minor reductions attributed to partial saturation of adsorption sites and slight structural changes. For BSP-A, adsorption capacities exceeded 294 mg/g in the first two cycles, slightly declined to 276 mg/g after four cycles, and stabilized at 238 mg/g after five cycles. Similarly, for 4-CL, adsorption capacities remained above 352 mg/g for the first two cycles, above 320 mg/g after four cycles, and at 298 mg/g after five cycles. These values demonstrate that Pyh/AC maintains high pollutant removal efficiency even after repeated regeneration, highlighting its structural robustness and long-term usability. The gradual decrease in performance can be linked to the potential blocking or deactivation of some adsorption sites due to residual pollutants, as well as minor textural and chemical modifications that may occur during multiple drying and rehydration processes. This behavior might be also assigned to that some adsorption sites become less accessible due to residual accumulation or weakening of secondary interactions.

The sustained adsorption efficiency of Pyh/AC across multiple cycles can be attributed to reversible physical adsorption mechanisms and the retention of its functional groups. Additionally, the porous structure and nitrogen-rich functional groups of polypyrrole hydrogel play a critical role in maintaining adsorption performance by enhancing the interaction between Pyh/AC and the target pollutants. The practical implications of Pyh/AC’s recyclability are significant for industrial wastewater treatment and large-scale environmental remediation projects. The ability to reuse the material multiple times reduces operational costs associated with adsorbent replacement, making Pyh/AC a highly economical alternative to traditional single-use adsorbents. Moreover, the water-based regeneration method eliminates the need for chemical-intensive desorption processes, reducing secondary pollution, regeneration cots, and making Pyh/AC a more sustainable solution for wastewater purification.

#### 3.2.8 Comparative studies

The adsorption performance of the Pyh/AC composite for bisphenol-A (BSP-A) and 4-chlorophenol (4-CL) was systematically compared with a range of adsorbents from the literature (Table 4). The results demonstrate the superior adsorption capacity of Pyh/AC, highlighting its effectiveness in phenol removal and its potential for practical applications in wastewater treatment. The Pyh/AC composite exhibited a maximum adsorption capacity (Qmax) of 321.4 mg/g for BSP-A and 365.8 mg/g for 4-CL, outperforming several conventional adsorbents. Compared to activated carbon, which is a widely used commercial adsorbent, Pyh/AC demonstrated double the adsorption capacity for BSP-A (158.7 mg/g for activated carbon vs 321.4 mg/g for Pyh/AC). Similarly, its capacity for 4-CL removal exceeded that of single-walled carbon nanotubes (334.34 mg/g), mesoporous carbon (333.33 mg/g), and activated carbon modified with amine groups (316.1 mg/g). These results confirm the composite’s remarkable ability to adsorb phenolic pollutants efficiently. The Pyh/AC composite outperformed advanced nanomaterials such as carbon nanofibers (63.3 mg/g for BSP-A) and graphene oxide (137 mg/g for BSP-A). It also surpassed bio-based adsorbents such as pinecone-activated ZnCl_2_ (73.53 mg/g) and alkali-modified biochar (71.4 mg/g). Even in comparison to functionalized adsorbents, such as organoclays (94.17 mg/g) and bentonite-chitosan composites (27.35 mg/g), the Pyh/AC composite exhibited significantly higher adsorption capacities. The comparative study highlights the exceptional performance of the Pyh/AC composite as an advanced adsorbent for BSP-A and 4-CL removal. Its superior adsorption capacity, cost-effective synthesis, and sustainability position it as a promising material for large-scale applications in water treatment and environmental remediation.

**TABLE 4:** Comparison between the adsorption performances of Pyh/AC composite and other materials in literature.

Adsorbents	Q_max_ (mg/g)	References
Bisphenol-A		
HCNTs/Fe_3_O_4_	113	Zhuang et al. (2024)
Activated carbon	158.7	Gao et al. (2023)
Carbon nanofibers	63.3	Gao et al. (2023)
Graphene oxide	137	Gao et al. (2023)
The walnut shell activated carbon (AC-Ws)	238.63	Rani et al. (2024)
Pinecones activated ZnCl_2_	73.53	Yazid et al. (2024)
Pinecones activated H_3_PO_4_	217.39	Yazid et al. (2024)
Alkali modified biochar	71.4	Jari et al. (2024)
CTAB/carboxymethyl cellulose/bagasse cryogels	31.7	Tang et al. (2021)
FA/HMBA/GP	302.7	Meneses et al. (2021)
bentonite-chitosan composite	27.35	Rind et al. (2025)
Organoclay	94.17	Ahari et al. (2024)
Pyh	241.8	This study
Pyh/AC	321.4	This study
4-Chlorophenol		
Silica-ionic liquid composite	87.58	De Farias et al. (2021)
MagneticFe_3_O_4_/activated carbon	128.2	Zhou et al. (2023)
MPHAC	183.64	Marwani and Bakhsh (2017)
Activated carbon derived from milk vetch	87	Zhang et al. (2015)
Activated carbon from aloe vera green wastes	47.6	Duan et al. (2020)
Nitrogen-doped carbon nanofibers (NCNFs)	427.3	Hadi et al. (2020)
Single walled carbon nanotubes	334.34	Noorimotlagh et al. (2015)
Nanosized activated carbon from bamboo and trees	427.3	Hadi et al. (2020)
CLDH/SWCNT nanocomposite	255.6	Omidi-Khaniabadi et al. (2015)
Activated pine sawdust-activated carbon	77.3	Gao et al. (2020b)
Activated carbon modified with amine groups	316.1	Madannejad et al. (2017)
Mesoporous carbon	333.33	Noorimotlagh et al. (2015)
Nanoporous graphene	125.01	Noorimotlagh et al. (2015)
nanosized activated carbons (NACs)	220	Zhang et al. (2019)
Pyh	284.3	This study
Pyh/AC	365.8	This study

## 4 Conclusions

Rice husk was used in the preparation of activated carbon with a graphitic structure and functionalized with polypyrrole hydrogel as a potential hybrid adsorbent (Pyh/AC) for bisphenol-A (BSP-A) and 4-chlorophenol (4-CL) pollutants. The composite exhibits strong adsorption performances, attaining 321.4 mg/g for BSP-A and 365.8 mg/g for 4-CL. These capacities are better than the hydrogel, which is experimentally related to the porosity, surface area, and functional groups of the activated carbon substrate. Theoretically, the active sites density as a steric factor confirmed the previous finding and explained the enhanced activities of Pyh/AC (169.7 mg/g (BSP-A) and 119.5 mg/g (4-CL)), as they are higher than the marked values for Pyh (110.5 mg/g (BSP-A) and 99.3 mg/g (4-CL)). The uptake occurred mainly by a multi-molecular process for the two phenolic compounds, which were adsorbed vertically, considering the adsorption capacity of each existing site (n = about 3 (BSP-A) and 6 (4-CL) molecules). The reactions were described according to the traditional Langmuir isotherm model and pseudo-first-order kinetics. The controlling mechanisms were identified as mainly physical processes based on the adsorption energy investigation, either from classic (<11 kJ/mol) or advanced (<20 kJ/mol) isotherm studies. The uptake behaviors show thermodynamically stable properties and display exothermic, favorable, and spontaneous properties.

## 5 Recommendation

Further studies will be conducted to functionalize and modify the structure with different chemical groups (sulfonic and amine groups) in addition to its integration in composites with other materials as strategies to enhance its adsorption efficiency, selectivity, and regeneration efficiency. Moreover, further studies will involve investigation of the composite in fixed bed column studies and realistic remediation of raw polluted water.

## Data Availability

The original contributions presented in the study are included in the article/Supplementary Material, further inquiries can be directed to the corresponding author.
